# Evaluating the Lower Urinary Tract Dysfunction Research Network Symptom Index-29 and SI-10 for Lower Urinary Tract Symptom Assessment Against with the International Prostate Symptom Score: A Cross-Cultural Validation and Agreement Study

**DOI:** 10.5152/tud.2026.26031

**Published:** 2026-06-19

**Authors:** Vittaya Jiraanankul, Phatsinee Likitpanpisit, Thanisorn Pattanasuwon, Weerayut Wiriyabanditkul, Sarayut Kanjanatarayon, Nattapong Binsri, Satit Siriboonrid

**Affiliations:** Division of Urology, Department of Surgery, Phramongkutklao Hospital, Bangkok, Thailand

**Keywords:** Lower urinary tract symptoms, Patient-Reported Outcome Measures, psychometrics, reproducibility of results, translations

## Abstract

**Objective:**

Lower urinary tract symptoms (LUTS) impose a significant burden on quality of life. Currently, no validated Thai version of the Lower Urinary Tract Dysfunction Research Network Symptom Index-29 (LURN SI-29) or its short form (SI-10) exists. Unlike the International Prostate Symptom Score (IPSS), LURN instruments comprehensively assess incontinence, pain, urgency, voiding, and nocturia across sexes. The study aimed to translate the LURN SI-29 and SI-10 into Thai and to evaluate their psychometric properties.

**Methods:**

A prospective, cross-sectional study was conducted involving 137 patients with LUTS at a tertiary hospital in Thailand. Translation was performed in accordance with Patient-Reported Outcome Consortium guidelines. Internal consistency (Cronbach’s alpha), test–retest reliability (intraclass correlation coefficient (ICC)), concurrent validity against the Thai IPSS, discriminant validity, and clinical agreement (Bland–Altman analysis).

**Results:**

The Thai LURN SI-29 and SI-10 demonstrated excellent internal consistency (*α* = 0.94 and 0.85) and test-retest reliability (ICC = 0.84 and 0.82), which remained robust across all educational levels. Both instruments correlated strongly with the IPSS total score (*ρ* = 0.85 and 0.80; *P* < .001). Furthermore, median LURN-SI scores increased significantly stepwise across IPSS mild, moderate, and severe grades (*P* < .001). Exploratory domain-level analysis suggested possible sex-related symptom patterns, with men scoring higher on voiding difficulties, while women exhibited greater incontinence and pelvic pain.

**Conclusion:**

The Thai LURN SI-29 and SI-10 are valid, reliable, and culturally appropriate. By capturing a broader symptom domain not included in the IPSS, they may support a more comprehensive LUTS evaluation. The SI-10 is ideal for rapid screening, while the SI-29 facilitates detailed assessment.

**Main Points**

- The Thai versions of the Lower Urinary Tract Dysfunction Research Network Symptom Index-29 (SI-29) and its short form (SI-10) demonstrated good reliability and robust validity, with strong internal consistency (Cronbach’s *α* = 0.94 and 0.85, respectively) and good test–retest stability (intraclass correlation coefficient > 0.80) regardless of educational level.
- Both instruments showed strong concurrent validity with the IPSS (Spearman ρ = 0.85 and 0.80, respectively) and effectively discriminated across International Prostate Symptom Score (IPSS) severity grades, confirming their ability to stratify clinical disease severity.
- The LURN SI-29 captures symptom domains not included in the IPSS, including incontinence and pain. Exploratory domain-level findings suggested possible sex-related symptom patterns, but these require confirmation in larger cohort studies.
- The LURN SI-10 is suitable for rapid outpatient screening, while the LURN SI-29 is recommended for detailed phenotyping in complex cases.

## Introduction

Lower urinary tract symptoms (LUTS) have become increasingly prevalent globally due to the aging population.^1^ The International Continence Society (ICS) categorizes LUTS into storage, voiding, and post-micturition symptoms, all of which can impair quality of life (QoL), sleep, and social functioning.^2^^,^^3^ Recent evidence also highlights the economic burden of these symptoms, particularly overactive bladder, which is associated with reduced workplace productivity and work hours.^4^ Accurate symptom assessment is the cornerstone of effective LUTS management. However, in many cultures, including Thailand, discussing urinary symptoms can be embarrassing for patients, leading to difficulties in history taking and potential assessment bias. Therefore, patient-reported outcome (PRO) measures are essential for accurate symptom assessment and for aligning treatment goals with patients.^5^

For decades, the International Prostate Symptom Score (IPSS) has been the most widely used tool for LUTS assessment. However, the IPSS was originally designed for men with benign prostatic hyperplasia (BPH), limiting its applicability to women and failing to assess key symptoms such as incontinence and pain.^6^ To address these limitations, the Symptoms of Lower Urinary Tract Dysfunction Research Network (LURN) developed the LURN Symptom Index-29 (LURN SI-29) and its short form, LURN SI-10. These tools assess domains including incontinence, pain, voiding, urgency, and nocturia, providing a detailed symptom profile to guide monitoring and treatment in both men and women.^7^^,^^8^

Despite its clinical utility, the LURN SI-29 has not been validated in the Thai language, limiting its adoption in clinical practice. Language-adapted questionnaires are essential not only for accurate comprehension but also for cultural acceptability, particularly in settings where discussing urinary symptoms carries social stigma.^5^ The EAU guidelines strongly recommend using validated PRO measures for LUTS assessment.^9^ This study aimed to translate the LURN SI-29 and SI-10 into Thai and to evaluate their psychometric properties—including reliability, content validity, and concurrent validity against the IPSS—in a Thai urological outpatient population.

## Material and Methods

The translation of the LURN-SI questionnaires was initiated in July 2025. The study protocol was approved by the Institutional Review Board of the Royal Thai Army Medical Department (S063q/68_Exp) on August 29, 2025. Prospective participant recruitment and data collection were subsequently conducted from September 2025 to April 2026 at a tertiary hospital.

### Patient Population

Patients aged 18 years or older presenting with LUTS were eligible for inclusion. Participants were also required to be able to self-administer the questionnaire in Thai. Patients were excluded if they had cognitive impairment, an active urinary tract infection, or gross hematuria. Additionally, patients who failed to complete both the baseline and retest questionnaires were excluded from the final analysis. Sample size was estimated based on Bonett's formula for Cronbach’s alpha, targeting a minimum coefficient of 0.70 with 90% power and a two-sided *α* of 0.05, yielding a minimum requirement of 86 participants. Enrollment continued beyond this threshold until the end of the study period to maximize statistical power, resulting in 137 participants. Written informed consent was obtained from all participants prior to enrollment.

### Tools for Data Collection

Baseline demographic and clinical characteristics were collected, including age, sex, body mass index (BMI), education level, and underlying comorbidities.

1. IPSS: The validated Thai version of the IPSS was used as the reference instrument for assessing concurrent validity.^10^ The IPSS consists of 7 symptom-related questions and 1 QoL question. The total symptom score ranges from 0 to 35 and classifies LUTS severity into 3 grades: mild (0-7), moderate (8-19), and severe (20-35). This severity grading was utilized to evaluate the validity of the Thai LURN tools.
2. LURN SI-29 and SI-10 (Thai Version): The LURN SI-29 assesses frequency and severity of LUTS across 29 items. The total score ranges from 0 to 100, with 0 reflecting the lowest symptom severity and 100 the most severe. The LURN SI-10 is a 10-item subset designed for rapid clinical screening with a maximum score of 38. Both tools were translated into Thai following the standardized forward-backward translation protocol described below.

### Linguistic Validation

Translation and cultural adaptation followed the consensus-based best practices recommended by the PRO Consortium.^11^ Two bilingual translators, both native Thai speakers fluent in English, independently produced forward translations of the LURN SI-29 and SI-10. A consensus meeting reconciled discrepancies into a single preliminary Thai version. A native English speaker, blinded to the original instrument, then back-translated the Thai version into English; any conceptual discrepancies identified were resolved by the research team. The preliminary Thai version was subsequently piloted with 10 Thai-speaking patients with LUTS to assess clarity and cultural appropriateness, and minor wording adjustments were made based on their feedback.

### Statistical Analysis

Data were analyzed using SPSS version 27.0 (IBM Corp., Armonk, N.Y., USA). Continuous variables with non-normal distributions, including LURN SI-29, SI-10, and IPSS scores, are summarized using medians and interquartile ranges. Differences in symptom scores between male and female participants were analyzed using the Mann–Whitney *U*-test. A *P* value of < .05 was considered statistically significant.

Content validity was assessed using the Item Objective Congruence index. Five experienced urologists actively involved in patient care independently rated each translated item using a 3-point scale: −1 = not congruent, 0 = uncertain, and +1 = congruent. The IOC for each item was calculated by summing the expert ratings and dividing by the number of experts. An IOC value ≥0.60 was considered acceptable for item retention.

The internal consistency of each symptom domain was assessed using Cronbach’s alpha coefficient (α). Cronbach’s *α* values were interpreted based on standard criteria: excellent (*α* ≥ 0.9), good (0.9 > *α* ≥ 0.8), and acceptable (0.8 > *α* ≥ 0.7). A value of ≥0.7 was considered the threshold for acceptable internal consistency. Test-retest reliability was evaluated by repeating the questionnaire at a 2- to 3-week interval to determine the intraclass correlation coefficient (ICC) based on a 2-way mixed effects model.

Given the non-normal distribution of symptom scores, Spearman’s rank correlation coefficient (*ρ*) was calculated to assess the concurrent validity between the LURN SI-29 and SI-10 scores and the IPSS total score. The Kruskal–Wallis test was then used to evaluate discriminant validity by comparing both LURN SI scores across the 3 IPSS severity grades (mild, moderate, and severe). For Bland–Altman analysis, raw scores were transformed to a 0-100 scale using the formula: standardized score = raw score/maximum possible score × 100. The IPSS was standardized as IPSS/35 × 100, and the LURN SI-10 was standardized as LURN SI-10/38 × 100. The LURN SI-29 total score was already expressed on a 0-100 scale.

## Results

### Demographic Characteristics

The study included 137 participants with a mean age of 65.9 ± 11.9 years. The cohort was predominantly male (84.7%, n = 116), with males being older than females (67.6 ± 11.6 vs. 56.5 ± 9.2 years). The largest proportion of participants had an education level below a bachelor’s degree (47.4%), followed by a bachelor’s degree (32.1%) and postgraduate education (20.1%). The prevalence of comorbidities was high in this population. Hypertension was the most common condition (56.9%), followed by dyslipidemia (45.3%), diabetes mellitus (19.0%), and heart disease (16.1%). Among male participants, 56.0% (n = 65) had a diagnosis of BPH. Based on IPSS severity grading, 31.4% of participants had mild symptoms, 54.7% had moderate symptoms, and 13.9% presented with severe symptoms (Table 1).

**Figure 1.**
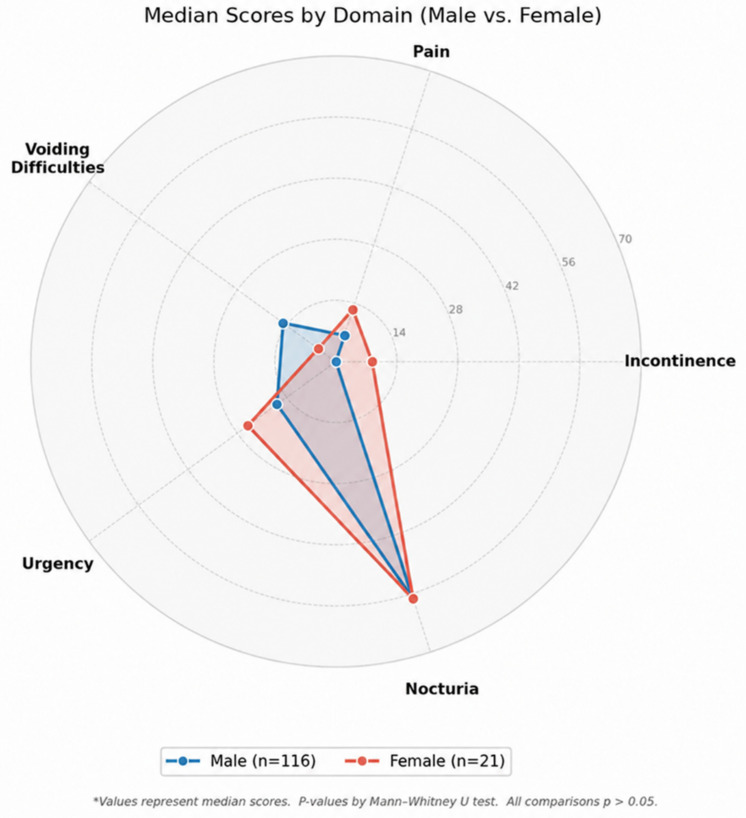
Radar plot of Lower Urinary Tract Dysfunction Research Network Symptom Index-29 (LURN SI-29) subdomain median scores by sex (male n = 117, blue; female n = 20, red). Men scored higher on voiding difficulties (15.0 vs. 5.0); women scored higher on incontinence (8.3 vs. 0.0), pain (12.5 vs. 6.3), and urgency (25.0 vs. 16.7). Nocturia was equivalent between sexes (57.1 vs. 57.1). No statistically significant differences were observed across any subdomain (all *P* > .05, Mann–Whitney *U*-test).

Despite comparable total symptom burden between sexes, exploratory domain-level analysis suggested distinct symptom trends (Table 2). Men scored higher on the Voiding Difficulties subdomain (median 15.0 vs. 5.0), consistent with obstructive physiology common in BPH. Women, by contrast, had higher median scores for incontinence (8.3 vs. 0.0), pain (12.5 vs. 6.3), and urgency (25.0 vs. 16.7), reflecting storage and sensory predominance. These differences did not reach statistical significance, primarily due to the limited statistical power of the small female subgroup (n = 21). Nevertheless, the directional consistency of these exploratory findings with established sex-specific LUTS phenotypes highlights the potential value of using a comprehensive tool such as the LURN SI-29 to capture a broader spectrum of symptoms. The sex-specific subdomain profiles are further illustrated in Figure 1.

### Reliability Analysis

Item-level IOC values ranged from 0.6 to 1.0, indicating acceptable content validity across translated items. Items requiring minor wording refinement were revised after expert review and patient pilot testing. Detailed item-level IOC values are shown in Supplementary Table 1.

The Thai LURN SI-29 showed high internal consistency, with a total Cronbach’s alpha of 0.94 and domain-specific alpha values ranging from 0.89 (Incontinence) to 0.75 (Nocturia). Test–retest reliability was assessed in 137 participants who completed both baseline and retest questionnaires. The ICC for LURN SI-29 was 0.84 (95% CI: 0.76-0.89), indicating good stability over the 2-3 week interval. Similarly, the LURN SI-10 demonstrated excellent reliability, with a Cronbach’s *α* of 0.85 and a test–retest ICC of 0.82 (95% CI: 0.76-0.87), confirming its reliability as a short screening tool (Table 3).

To evaluate the effect of educational background on test-retest reliability, participants were divided into 3 groups: below bachelor’s degree, bachelor’s degree, and postgraduate degree. The analysis revealed excellent reliability across all educational levels. ICC values were 0.83 for the below bachelor’s group (95% CI: 0.70-0.90), 0.84 for the bachelor’s degree group (95% CI: 0.71-0.91), and 0.86 for the postgraduate degree group (95% CI: 0.72-0.93). ICC estimates were similar across educational strata and showed overlapping 95% CIs. Because no formal statistical comparison of ICCs was performed, these findings should be interpreted descriptively. Overall, these results suggest that the Thai LURN-SI maintains stable test–retest reliability across respondents with different educational backgrounds.

### Concurrent and Agreement Validity (with IPSS)

**Figure 2.**
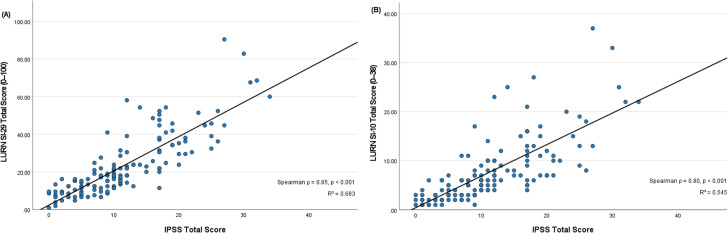
Scatter plots illustrating concurrent validity between the International Prostate Symptom Score (IPSS) total score and the Thai Lower Urinary Tract Dysfunction Research Network (LURN) Symptom Index (n = 137). Solid lines denote fitted linear regression lines. (A) IPSS vs. LURN SI-29: Spearman ρ = 0.85, *P* < .001; *R*^2^ = 0.683. (B) IPSS vs. LURN SI-10: Spearman ρ = 0.80,* P* < .001; *R*^2^ = 0.545. The higher *R*^2^ for the SI-29 reflects its greater content coverage relative to the short form.

Total scores for the LURN SI-29 and SI-10 correlated strongly with the IPSS total score using Spearman’s rank correlation (ρ = 0.85 and ρ = 0.80, respectively; both *P* < .001; Figure 2). Discriminant validity was confirmed using the Kruskal–Wallis test, which revealed statistically significant differences in median LURN SI-29 and SI-10 scores across the 3 IPSS severity groups (*P* < .001), with scores increasing stepwise across mild, moderate, and severe groups (Table 4).

**Figure 3.**
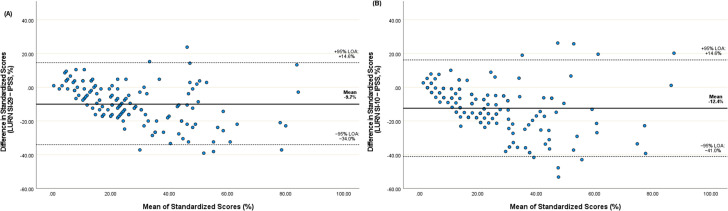
Bland–Altman plots assessing agreement between the Thai Lower Urinary Tract Dysfunction Research Network (LURN) Symptom Index and the International Prostate Symptom Score (IPSS), with all instruments standardized to a 0–100 scale (n = 137). The solid line denotes mean bias; dashed lines represent 95% limits of agreement (LOA). (A) LURN SI-29 vs. IPSS: mean bias −9.7% (95% LOA: −33.6% to +14.2%). (B) LURN SI-10 vs. IPSS: mean bias −12.4% (95% LOA: −41.1% to +16.2%). Proportional bias is evident in both panels, with the IPSS yielding progressively higher relative scores at greater symptom severity, indicating the 2 instruments are not directly interchangeable.

To assess agreement, scores of the LURN SI-29, SI-10, and IPSS were adjusted to a 0–100 scale using the formula described above. Bland–Altman analysis revealed a mean bias of −9.7% (95% limit of agreement (LOA): −33.6% to +14.2%) for the LURN SI-29 and −12.4% (95% LOA: −41.1% to +16.2%) for the LURN SI-10 compared to the IPSS (Figure 3). This indicates that both LURN SI-29 and SI-10 yield generally lower standardized scores than IPSS. Visual inspection suggests proportional bias: while agreement is reasonable for mild-to-moderate symptoms, the IPSS reports increasingly higher relative scores than the LURN as symptom severity worsens. This suggests that in severe cases, the IPSS tends to report a higher relative score compared to the LURN-SI-29.

## Discussion

This study provides evidence of reliability and concurrent validity of the Thai versions of the LURN SI-29 and SI-10 in assessing LUTS. The results align with the original validation study in the United States and a recent validation in Türkiye, supporting the global use of LURN questionnaires.^7^^,^^12^ Strong content validity (IOC 0.6-1.0) and excellent internal consistency (*α* > 0.90 for SI-29) establish the Thai version's conceptual equivalence and suitability for local clinical practice.

Both instruments demonstrated high internal consistency. The Thai LURN SI-29 achieved an excellent Cronbach’s alpha of 0.94, and the LURN SI-10 also demonstrated strong consistency with an *α* of 0.85. While the nocturia subdomain had the lowest internal consistency (0.75), it remains well above the acceptable threshold of 0.70, confirming the reliability of all subdomains for clinical application. Test-retest reliability was consistent (ICC > 0.8) and unaffected by participants’ educational levels. This finding is important for public hospitals serving a wide range of socioeconomic populations, suggesting that Thai LURN-SI performs consistently across educational levels.

Concurrent validity with the IPSS was strong (*ρ* > 0.8), demonstrating that the LURN instruments effectively measure symptom severity consistent with established questionnaires. The significant association with IPSS severity grades supports the ability of the LURN SI to stratify patients into clinically meaningful severity groups. Median LURN SI-29 scores progressively increased from 9.5 in mild IPSS to 44.8 in severe IPSS (*P* < .001), confirming excellent discriminant validity. This suggests that the Thai LURN SI tools are sensitive enough to categorize disease severity in a manner consistent with established clinical standards.

Despite strong concurrent validity, Bland-Altman analysis demonstrated that the LURN SI and IPSS are not directly interchangeable. Both LURN instruments yielded systematically lower standardized scores than the IPSS, with mean biases of −9.7% for the SI-29 and −12.4% for the SI-10. This finding is mechanistically expected, as patients without incontinence or pelvic pain score 0 across those subdomains, which lowers their total LURN score relative to the IPSS. A proportional bias was also apparent, and the gap between the 2 instruments widened at higher symptom severity. These differences likely reflect variations in item content and domain symptoms weighting between the instruments. Specifically, the IPSS focuses on voiding and storage symptoms, whereas the LURN-SI includes additional domains such as incontinence and pain. Therefore, these findings support the complementary use of instruments rather than the superiority of one over the other. The narrower limits of agreement for the SI-29 versus SI-10 confirm that the short form trades precision for efficiency, as expected of a screening instrument.

A key advantage of the LURN SI highlighted by the study is the ability to detect symptoms often not assessed by the IPSS, specifically incontinence and pain. This capability is clinically important for subtyping LUTS at the individual patient level. The exploratory analysis suggested distinct symptom patterns: women predominantly presented with storage and pain symptoms, while men were more affected by voiding difficulties. By detecting these specific symptoms, the LURN SI supports more precise phenotyping of the patient's condition, especially for women whose symptoms are not assessed by the IPSS.^8,13,14^

Based on these findings, a two-tiered clinical approach is proposed: the LURN SI-10 for rapid symptom screening in general urology outpatient clinics, and the LURN SI-29 for detailed phenotyping in patients with complex presentations or refractory symptoms. This approach balances clinical efficiency with diagnostic thoroughness, ensuring that patients with specific dominant symptoms such as urgency-predominant overactive bladder, stress incontinence, or pelvic pain receive phenotype-directed treatment rather than empirical therapy. Importantly, both tools apply equally to male and female patients, offering a meaningful advantage over the IPSS in a mixed-sex clinical setting.

This study has several limitations. First, the gender imbalance in the study (84.7% male) reflects the military hospital setting and limits the statistical power of sex-based comparisons; observed sex-specific symptom profiles should therefore be interpreted as exploratory trends. Second, symptom fluctuations or minor lifestyle changes during the 2–3 week test-retest interval could not be strictly controlled. Third, the sample size (N = 137) was calculated primarily for internal consistency, and the participant-to-item ratio was insufficient for confirmatory factor analysis. Finally, using the IPSS as the sole comparator limits specific validation for domains like incontinence and pain. Future multicenter studies should employ larger, gender-balanced cohorts, perform confirmatory factor analysis, and incorporate specific comparators (e.g., International Consultation on Incontinence Questionnaire Female Lower Urinary Tract Symptoms Modules (ICIQ-FLUTS)/International Consultation on Incontinence Questionnaire Male Lower Urinary Tract Symptoms Module (ICIQ-MLUTS), Overactive Bladder Symptom Score (OABSS), and voiding diaries).

## Conclusion

The Thai versions of the LURN SI-29 and SI-10 are valid, reliable, and culturally appropriate instruments for assessing LUTS in Thai-speaking patients. Both tools demonstrate strong concurrent validity with the IPSS and effectively discriminate across severity grades. Unlike the IPSS, they apply to patients of both sexes and capture a broader symptomatic profile, including incontinence and pain, which may support more comprehensive LUTS evaluation. These instruments are ready for adoption in Thai urological practice. Future studies should evaluate their responsiveness to treatment, which will determine their utility for longitudinal monitoring of clinical outcomes.

## Supplementary Materials

Supplementary Material

## Figures and Tables

**Table 1.** Demographic and Baseline Clinical Characteristics of the Study Population (n = 137)

Characteristics	Total (137)	Male (116)	Female (21)
Age, years (mean ± SD)	65.9 ± 11.9	67.6 ± 11.6	56.5 ± 9.2
BMI	24.9 ± 3.6	24.9 ± 3.3	25.0 ± 4.9
Education level			
Below bachelor	65 (47.4)	54 (46.6)	11 (52.4)
Bachelor	44 (32.1)	37 (31.9)	7 (33.3)
Postgraduate	28 (20.1)	25 (21.6)	3 (14.3)
Comorbidities			
Hypertension	78 (56.9)	69 (59.5)	9 (42.9)
Diabetes mellitus	26 (19.0)	23 (19.8)	3 (14.3)
Dyslipidemia	62 (45.3)	55 (47.4)	7 (33.3)
Heart disease	22 (16.1)	21 (18.1)	1 (4.8)
BPH (male only)	65 (56.0)	65 (56.0)	–
IPSS severity grading			
Mild (0-7)	43 (31.4)	34 (29.3)	9 (42.9)
Moderate (8-19)	75 (54.7)	66 (56.9)	9 (42.9)
Severe (20-35)	19 (13.9)	16 (13.8)	3 (14.3)

BMI, body mass index; BPH, benign prostatic hyperplasia.

**Table 2.** Comparison of Median (IQR) LURN SI-29 and SI-10 Scores Between Male and Female Participants

Domain / Scale	Total (n = 137)	Male (n = 116)	Female (n = 21)	P*
LURN SI-29	18.1 (11.4-31.4)	18.1 (11.4-30.5)	16.2 (12.9-37.14)	.77
Incontinence	0.0 (0.0-12.5)	0.0 (0.0-12.5)	8.3 (0.0-27.1)	.18
Pain	6.3 (0.0-18.8)	6.3 (0.0-18.8)	12.5 (0.0-34.4)	.17
Voiding difficulties	15.0 (0.0-30.0)	15.0 (5.0-32.5)	5.0 (0.0-32.5)	.10
Urgency	16.7 (0.0-33.3)	16.7 (0.0-33.3)	25.0 (8.3-50.0)	.18
Nocturia	57.1 (42.9-85.7)	57.1 (42.9-85.7)	57.1 (28.6-85.7)	.88
LURN SI-10	6.0 (3.0-10.0)	6.0 (3.0-10.0)	6.0 (4.0-9.5)	.92
IPSS	10.0 (6.0-17.0)	10.5 (7.0-17.0)	9.0 (4.0-18.5)	.60

^*^P values calculated using Mann–Whitney *U*-test.

IQR, interquartile range; LURN SI, Lower Urinary Tract Dysfunction Research Network Symptom Index.

**Table 3.** Reliability Analysis: Internal Consistency (Cronbach’s alpha) and Test–Retest Reliability (ICC)

**Domain/Scale**	**No. of Items**	**Internal Consistency (Cronbach’s *α*)**	Test–Retest Reliability (ICC) [95% CI]*
LURN SI-29	28	0.937	0.836 (0.758-0.887)
Incontinence	6	0.893	0.844 (0.787-0.887)
Pain	4	0.858	0.783 (0.674-0.853)
Voiding difficulties	5	0.861	0.776 (0.700-0.835)
Urgency	3	0.84	0.736 (0.641-0.807)
Nocturia	2	0.749	0.736 (0.648-0.804)
LURN SI-10	10	0.847	0.824 (0.757-0.872)

^*^Intraclass correlation coefficient (ICC) (two-way mixed model, absolute agreement).

LURN SI, Lower Urinary Tract Dysfunction Research Network Symptom Index.

**Table 4.** Comparison of Median (IQR) LURN Scores Across IPSS Severity Grades

Scale	Mild (n = 43)	Moderate (n = 75)	Severe (n = 19)	P*
LURN-SI-29	9.5 (6.7-13.3)	21.9 (16.2-31.4)	44.8 (32.4-60.0)	<.001
LURN-SI-10	3.0 (2.0-4.0)	7.0 (5.0-10.0)	13.0 (10.0-22.0)	<.001

^*^Calculated using Kruskal–Wallis test.

IQR, interquartile range; LURN SI, Lower Urinary Tract Dysfunction Research Network Symptom Index.

## Data Availability

The data that support the findings of this study are available on request from the corresponding author.
