# Supplementary material for: Evaluating the Lower Urinary Tract Dysfunction Research Network Symptom Index-29 and SI-10 for Lower Urinary Tract Symptom Assessment Against with the International Prostate Symptom Score: A Cross-Cultural Validation and Agreement Study
Source: Urol Res Pract. 2026 Jun 19;52:e26031. doi: 10.5152/tud.2026.26031 (PMC13324903; doi:10.5152/tud.2026.26031)
Supplement: Supplementary Material [file supplementary_material.pdf]

Supplementary Table 1. Item Objective Congruence (IOC) evaluation of the Thai version of the LURN SI-29 Questionnaire by the expert panel (N=5).

Item	Domain	Brief Item Description	E1	E2	E3	E4	E5	Total	IOC	Interpretation
1	Incontinence	Completely lose control of bladder	1	1	1	1	1	5	1.0	Acceptable
2	Incontinence	Leak urine after feeling a sudden need	1	1	1	1	1	5	1.0	Acceptable
3	Incontinence	Leak urine while laughing, sneezing, or coughing	1	1	0	1	1	4	0.8	Acceptable
4	Incontinence	Leak urine when doing physical activities	1	1	1	1	1	5	1.0	Acceptable
5	Incontinence	Leak urine caused by walking at usual speed	1	0	1	1	1	4	0.8	Acceptable
6	Incontinence	Leak urine during the night (wetting bed/pad)	1	1	1	1	1	5	1.0	Acceptable
7	Pain	Pain or discomfort in bladder while filling	1	1	1	1	1	5	1.0	Acceptable
8	Pain	Pain or discomfort in bladder when full	1	1	1	1	0	4	0.8	Acceptable
9	Pain	Pain or discomfort while urinating	1	1	1	1	1	5	1.0	Acceptable
10	Pain	Pain or discomfort right after finishing urination	0	1	1	1	1	4	0.8	Acceptable
11	Voiding	Have to push when urinating	1	1	1	1	1	5	1.0	Acceptable
12	Voiding	Delay before started to urinate (Hesitancy)	1	1	1	1	1	5	1.0	Acceptable
13	Voiding	Urine flow stop and start again (Intermittency)	1	1	1	1	1	5	1.0	Acceptable
14	Voiding	Urine flow slow or weak	0	1	0	1	1	3	0.6	Acceptable
15	Voiding	Trickle or dribble at the end of urine flow	1	1	1	1	1	5	1.0	Acceptable
16	Urgency	Feel a sudden need to urinate	1	1	1	1	1	5	1.0	Acceptable
17	Urgency	Sudden need to rush to urinate for fear of leaking	1	1	0	0	1	3	0.6	Acceptable
18	Urgency	Difficulty waiting more than a few minutes	1	1	1	0	1	4	0.8	Acceptable
19	Nocturia	Number of times waking up to urinate at night	1	1	1	1	1	5	1.0	Acceptable
20	Nocturia	Waking up at least once due to need to urinate	1	1	1	1	1	5	1.0	Acceptable
21	Storage/Freq.	Number of times typically urinate during waking hours	1	1	1	1	1	5	1.0	Acceptable
22	Storage/Freq.	Time typically passed between urinations	1	0	1	1	1	4	0.8	Acceptable
23	Urgency	Typical urge to urinate when waking up at night	1	1	1	1	1	5	1.0	Acceptable
24	Storage/Freq.	Constant need to urinate that did not go away	1	1	1	1	1	5	1.0	Acceptable
25	Post-Mict.	Feeling that bladder was not completely empty	1	1	1	0	1	4	0.8	Acceptable
26	Post-Mict.	Dribble urine just after zipping pants / underwear	1	1	1	1	1	5	1.0	Acceptable
27a	Voiding	(Women only) Spraying or change in direction of stream	1	1	0	1	1	4	0.8	Acceptable
27b	Voiding	(Men only) Splitting or spraying of urine stream	1	1	1	1	1	5	1.0	Acceptable
28	Overall	Overall bother by urinary symptoms	1	1	1	1	1	5	1.0	Acceptable

E1–E5 = Expert 1 to Expert 5 (Board-certified urologists).
Scoring criteria: +1 = Congruent (Clearly measures the objective); 0 = Unsure (Unclear if it measures the objective); -1 = Incongruent (Does not measure the objective).
IOC Formula: Calculated as the sum of scores divided by the number of experts.
Interpretation Threshold: An IOC score of 0.6 is considered acceptable for content validity.
